# A Proxy Method to Bridge LCA Data Gaps Using Automated Material Classification and Probabilistic Under-Specification

**DOI:** 10.1021/acs.est.5c12282

**Published:** 2026-04-14

**Authors:** Ethan Ellingboe, Monica Huang, Azeezah Sultana Priyota, Kathrina Simonen

**Affiliations:** Department of Architecture, 7284 University of Washington, Seattle, Washington 98195, United States

**Keywords:** life cycle assessment, data gap, proxy data, chemical classification, novel materials, probabilistic under-specification

## Abstract

Life cycle assessments (LCAs) are essential for understanding the environmental impacts of material production. However, gaps in life cycle inventory (LCI) data for material and chemical inputs present a key challenge for LCA practitioners, especially in the early design stages. Strategies for filling in these gaps require additional time and expertise, which can hinder the LCA’s completion. This study combined automatic material classification and probabilistic under-specification to create a time-efficient method to fill material LCI data gaps. To illustrate the proposed method, proxy environmental impact distributions were generated using publicly available material LCI data classified into the *ChemOnt* chemical taxonomy using the open-source chemical classification software *ClassyFire*. Input materials with data gaps were then classified into the same taxonomy, where proxy environmental impact values could be selected from the available distributions to quickly fill in any data gaps. Although these methods were applied to classify material production processes available in the Federal LCA Commons and Ecoinvent databases, they can be applied to any LCA database. This study shows that classifying materials by their chemical structure produces taxonomies with increased granularity relative to industrial classification, improving the ability of under-specified proxy data to be used for differentiating the environmental impacts of competing designs.

## Graphical abstract

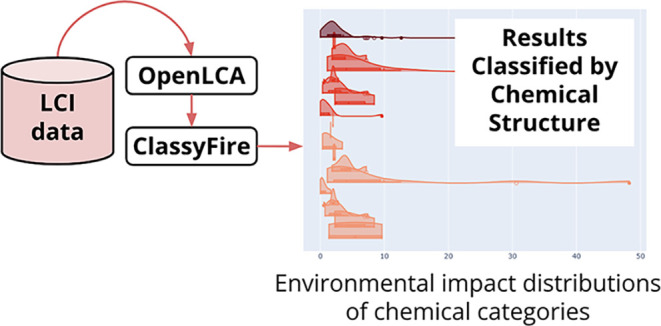

## 1 Introduction

### 1.1 Background

Evaluation of the environmental performance of materials and products relies on life cycle assessment (LCA), however, sparse LCA data are available for specialty chemicals and input materials, which are used in the development of novel materials, among other purposes. Filling data gaps for specialty materials and chemicals requires additional time, resources, and expertise, resulting in added complexity for novel material LCAs or omission within LCA studies.^1–3^ However, if gaps are unfilled, the completeness of the LCA model is decreased, resulting in potential underestimation of environmental impacts.^4^

Numerous strategies have been utilized for streamlining life cycle assessments to facilitate rapid LCAs, such as selecting “proxy” or “surrogate” data; interpolation or extrapolation of existing data; and process modeling.^5^ LCA streamlining approaches may be especially critical in early design stages, when time and resources are constrained. One strategy available to LCA practitioners to fill (or “bridge”) data gaps involves using proxy or surrogate data that are available for a similar input flow. Another strategy is probabilistic under-specification, which has been established in LCA literature as a promising method to estimate proxy environmental impact values.^1,5–14^

The applicability of under-specification methods in early-stage screening LCAs relies on the ability to distinguish the resulting environmental impact distributions of comparable designs in a meaningful or statistically significant way. Robust probabilistic methods to compare resulting environmental impact distributions of comparable designs have been proposed in previous under-specification literature.^6,9,10,12,16,17^ For example, the work by Tecchio evaluated coefficient of variation and median absolute deviation coefficient of variation (MAD–COV) to characterize dispersion of environmental impact distributions, and a comparison indicator calculation to compare environmental impact distributions of competing designs.^6^ Probabilistic triage methods have been implemented in various studies to prioritize inputs for further data specification by environmental impact value, coefficient of variation, or MAD–COV as in works by Tecchio;^6,10^ squared Spearman rank correlation coefficients as in works by Hester and AzariJafari;^12,16^ or Sobol sensitivity analysis as in the work by Alcaraz.^9^ The work by Torres implements kernel density estimation for the comparison of material environmental impact probability distribution functions.^17^ While application of probabilistic under-specification methods for filling data gaps is common for LCAs of buildings and building materials, the proposed methods may be applied in any LCA for which data gaps for material and chemical inputs exist.^4,9^

### 1.2 Toward Improved Proxy Data Granularity

When probabilistic under-specification methods are applied in practice, the underlying life cycle inventory or environmental impact data must be categorized into a material classification taxonomy, which enables sufficient differentiation of impact results for competing designs. If the selected material taxonomy is not sufficiently granular, producing under-specified environmental impact distributions with too much variance, differences in environmental impact results of competing designs may not be statistically distinguishable. In this case, further specification is needed to sufficiently resolve the environmental impacts resulting from the designs under consideration. As such, the usefulness of under-specification methods in early-stage LCA can benefit from greater granularity and depth (number of levels) in the material taxonomy employed.

Life cycle inventory databases typically use industrial classification systems for the categorization of the data. This is reasonable considering the wide range of processes contained in these databases, from electricity generation and transportation to waste handling. The Federal LCA Commons database uses the North American Industrial Classification System (NAICS), and the Ecoinvent database uses the International Standard Industrial Classification (ISIC) system. The default NAICS classification system in the Federal LCA Commons data considered in this study has two levels containing nine broad industrial categories in the first level (two-digit codes) and sixty-four specific industrial categories in the second level (four-digit codes). The default ISIC classification system in the Ecoinvent data considered in this study has four levels containing seven broad industrial “sections” in the first level (one-letter codes), twenty-three “divisions” in the second level (two-digit codes), fifty-six “groups” in the third level (three-digit codes), and 102 “classes” in the fourth level (four-digit codes). Note that additional Federal LCA Commons and Ecoinvent data exist, which are not considered in this study. Generally, all material and chemical production processes are classified under the “manufacturing” category at the first industrial classification level (“31–33: Manufacturing” in NAICS and “C: Manufacturing” in ISIC). Thus, considering only material manufacturing processes effectively reduces the NAICS classification system to a single level with twenty-three relevant subcategories and the ISIC classification system to three levels with six relevant manufacturing “divisions”, twelve “groups”, and twenty-three “classes”.

Incorporating the use of an open-source, chemical structure-based material classification system with probabilistic under-specification can provide a consistent, transparent, and reproducible method to calculate proxy environmental impact distributions for material categories at varying degrees of specification. In turn, these proxy impact distributions can be used to fill data gaps for input materials and chemicals in rapid screening LCAs during early-stage product development with greater specificity relative to industrial classification systems. The chemical structure-based taxonomy ChemOnt contains up to 11 taxonomic levels and >4800 material categories, exceeding the industrial classification systems described above in both depth and granularity for material and chemical classification.^15^

In this study, we propose utilizing a classification system based on a material’s chemical structure to categorize life cycle inventory and impact assessment data for material production supply chains. Improving the use of industrial classification systems, this approach for filling LCA data gaps in early design stages better identifies any available data for compositionally similar materials and increases the granularity of material categories available for probabilistic under-specification. The proposed framework for classifying existing material and chemical production data into a chemical structure-based taxonomy may be implemented in software tools, such as those demonstrated in the accompanying Supporting Information (SI), which enable any LCA practitioner to fill data gaps with estimates of environmental impacts that are transparent, reproducible, and informed by chemical structure.

## 2 Methods

The methods proposed in this paper are separated into four distinct stages described below. These include methods to evaluate environmental impacts from life cycle inventory data (Section 2.1), to automatically classify chemicals and materials into a structure-based chemical taxonomy (Section 2.2), to create statistical distributions of environmental impacts for taxonomic categories (Section 2.3), and apply the environmental impact distributions to fill data gaps for input chemicals and materials with no available life cycle inventory (LCI) or life cycle impact assessment (LCIA) data (Section 2.4). Figure 1 shows a high-level schematic of this framework, and a short description of how it can be used to generate environmental impact distributions for probabilistic under-specification methods is as follows:

(1) In the selected database and LCA software, existing LCI data for manufacturing processes with material or chemical outputs are transformed into product systems. LCIA results are evaluated for each product system using the selected LCIA method. In this work the *olca-ipc* python module was used to automate product system creation and LCIA calculation in OpenLCA.^18,19^

(2) Names of materials and chemicals produced from manufacturing processes with existing LCIA data are automatically searched for matching International Chemical Identifier (InChI) keys, in this study primarily using the PubChem Power User Gateway Representational State Transfer (PUG-REST) API.^20,21^ International Chemical Identifier keys (InChI keys) are then automatically classified into the ChemOnt taxonomy using the *Batch Compound Classification* tool.^15,22^

(3) Data for materials or chemicals are grouped by chemical classification, enabling statistical analyses of LCI or LCIA result distributions within chemical groups. The spreadsheet tool in the SI demonstrates calculation of minimum, 20th percentile, median, 80th percentile, and maximum environmental impact values; however, advanced statistical methods for probabilistic under-specification could also be implemented.
(4) Materials or chemicals with data gaps are then similarly matched to an InChI key and classified. Within the corresponding material classification, a single material with available data may be identified that matches the chemical composition of the material in question, or which is deemed sufficiently representative for use as a proxy. If no match is identified and no single proxy is sufficiently representative, the distribution of all material data within the material group may be used for probabilistic under-specification methods.

**Figure 1:**
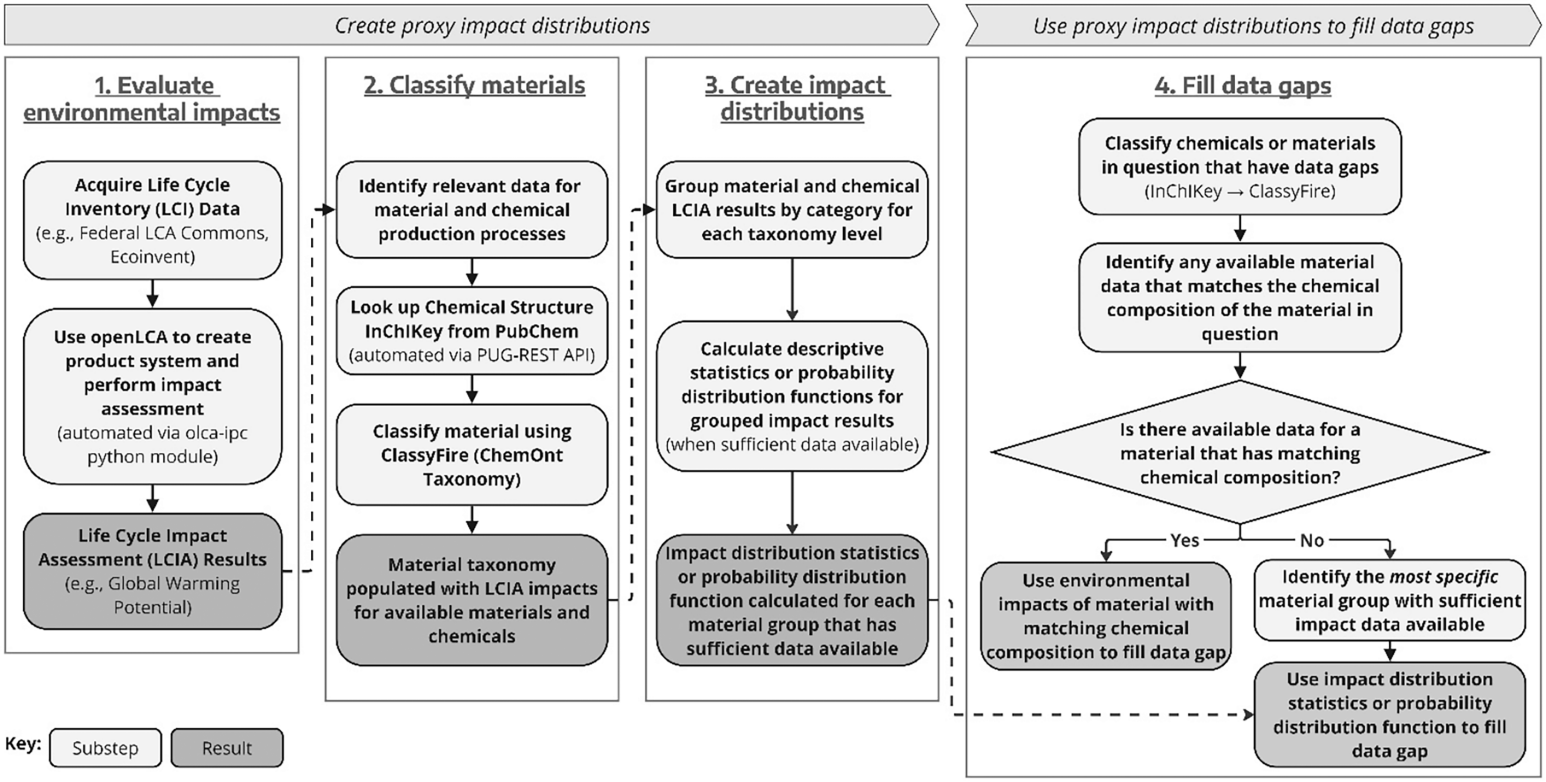
A high-level schematic illustrating the proposed framework for material classification and estimation of LCI or LCIA result distributions for use as proxy values or in probabilistic under-specification methods to fill data gaps.

The most specific group to which the material is classified should be considered first. If insufficient data exist in the most specific material group, the next broadest level in the taxonomy should be considered, and so on, until the most specific class having sufficient data available is identified.

### 2.1 Evaluation of Environmental Impacts

Evaluation of life cycle impact assessment (LCIA) results from life cycle inventory (LCI) data was conducted using the open-source LCA software OpenLCA (version 2.4.0).^18^ LCIA evaluation follows alternate procedures depending on whether the supply chain emissions have been aggregated (system process) or if emissions for each step unit process in the supply chain are linked to each other (unit process).^23^ For aggregated emissions, no supply chain creation is necessary, as the upstream and direct emissions for the supply chain have been aggregated into the reported emissions quantities. For unit process data, the supply chain is automatically linked in the creation of the product system based on the default provider process set for each technosphere flow in the database.

To enable rapid evaluation and screening of material and chemical inputs for novel material LCAs, a Python script using the openLCA-python API (olca-ipc) was developed to generate product systems and evaluate LCIA impacts for a large volume of process LCI data.^24–26^ While customization and fine-tuning of the upstream product system can improve the quality of an LCA model, the default provider linking and flow quantities in each product system were used for the evaluation of environmental impacts in this study. Additionally, some unit process LCI data may include flows that have no provider process available, resulting in an incomplete or “cutoff” flow in the product system. Completeness of process LCI data should be considered in the evaluation of data quality; completeness of environmental impact results depends on completeness of the process LCI data.

To demonstrate the methods and framework described herein, the five core impact assessment categories required by ISO 21930:2017 were selected for evaluation: 100-year global warming potential (GWP), acidification potential (AP), eutrophication potential (EP), ozone depletion potential (ODP), and photochemical oxidant creation potential (POCP). The US EPA ISO 21930-LCIA-US (v0.1) LCIA method was used to evaluate these impact categories for Federal LCA Commons data.^27^ GWP impacts were evaluated with IPCC AR5 characterization factors. Only GWP impact results are discussed below; however, classified impact results for AP, EP, ODP, and POCP are available in the SI.

### 2.2 Automated Material Classification

The structure-based chemical taxonomy ChemOnt was utilized as a consistent, transparent, and reproducible classification system for material and chemical production LCI data.^15^ Classifying materials and chemicals based on their chemical composition and structure provides a purely objective classification method and increases the depth and granularity of partitioning for chemical entities (relative to industrial classification systems). Only *chemical entities*, defined as “constitutionally or isotopically distinct atoms, molecules, ions, ion pairs, radicals, radical ions, complexes, conformers, etc., identifiable as a separately distinguishable entity”, can be classified in the ChemOnt taxonomy.

All processes representing the production of chemical entities were identified from the full list of process LCI data available in the Federal LCA Commons and Ecoinvent databases.^28–32^ The standard name of the chemical entity represented by each corresponding process was determined, which in turn was used to produce a consolidated list of all of the chemical entities represented in the LCI database. LCI processes representing production of materials and chemicals were identified by industrial classification in relevant manufacturing categories (e.g., ISIC division “20: Manufacturing of chemicals and chemical products”), then manually verified. Product material and chemical names were automatically separated from LCI process names using spreadsheet string parsing formulas and then manually verified.

For the materials and chemicals in the LCI database, the chemical name was interpreted from the LCI metadata. The international chemical identifier key (InChI key) was determined for each chemical entity represented in the database and used for classification.^21^ The public chemical information database PubChem was used as the primary source for chemical entity InChI keys. Use of programmatic retrieval tools for PubChem provided a rapid method for the InChI key based on chemical name. Please see the relevant article for more information related to programmatic retrieval of InChI keys, as well as the spreadsheet tool in the SI for an example use of the PubChem PUG-REST API for automatic InChI key lookup.^20^ In the case there was no entry or InChI key found through PubChem for a chemical entity in the LCI database, other sources were checked for the InChI key. If no InChI key was found in any source, then the Simplified Molecular Input Line Entry System (SMILES) structure was used for classification. If no InChI key or SMILES structure was available for a chemical, then it was not included as a chemical entity.

Once the InChI key or SMILES structure was identified for all chemical entities present in the LCI database, these were used to classify each chemical entity into the ChemOnt taxonomy using the open-source web tool built for this purpose, ClassyFire.^15^ The batch ClassyFire tool was used to classify large numbers of chemical entities.^22^ The data classification was originally completed by one team member, and a second team member performed spot checks to help ensure that the method was being applied consistently. The team found that the method was clear enough to obtain consistent results when it was applied by different individuals.

### 2.3 Creation of Statistical Distributions of Impacts

After classification, each chemical entity with LCI data for its production available in the database was assigned to a chemical category at each level of the hierarchical chemical taxonomy. The environmental impacts for each LCI were evaluated as described above. Subsequent grouping of environmental impact values according to chemical classification generated impact value distributions for each chemical category in the taxonomy that had more than one process available in the LCI database. If only one constituent process existed in a chemical category, then no impact distribution was created, but the deterministic impact values could be used as proxy values to fill data gaps if deemed sufficiently representative of the target material. Impact distributions for chemical entity categories were unweighted in this study.

To further explore the effect of including or removing outliers on the use of these methods, GWP impact outliers were identified for each chemical category using the Tukey method.^33^ Statistical distributions of environmental impact values were generated before and after the removal of outliers for comparison. These statistical distributions are subsequently used to fill data gaps in early-stage screening LCAs via under-specification of input chemicals and materials.

### 2.4 Filling Data Gaps with Probabilistic Under-Specification

To fill LCA data gaps using the impact value distributions generated through the methods described above, the target material or chemical with no available LCI data was classified into the same chemical taxonomy. Then, the most specific chemical category with available LCI data for other constituent entities to which the target entity belongs was used to estimate the environmental impacts of producing the target chemical or material. If no LCI data were available for any constituent chemical entities in the most specific category to which the target entity belongs, the category at the next hierarchical level in the taxonomy was checked, and so on until a category was reached with LCI data available for a sufficient number of constituents.

Deterministic proxy values may be selected from the impact value distributions for a category. Expert judgment can assist in the selection of deterministic proxy values from given distributions. For example, if it is known that production of the target material requires an energy-intensive processing step relative to the other material production processes represented in the proxy distribution, a higher proxy impact value may be selected.

## 3 Results

This section presents the GWP results of classifying the LCIA data from Federal LCA Commons and Ecoinvent into the ChemOnt taxonomy in various data visualizations (Section 3.1), and a case study of five materials demonstrates how this method can be used to fill in proxy data for specialty materials and chemicals (Section 3.2).

### 3.1 Material Classification and Environmental Impact Distributions

Applying the proposed methods to LCI data available in the Federal LCA Commons resulted in the identification and classification of 209 process LCIs representing the production of ninety-two unique chemical entities. Classification into the ChemOnt taxonomy assigned materials into two kingdoms, ten superclasses, thirty-one classes, and forty-six subclasses, and environmental impact distributions were generated for each. The default industrial classification of the same Federal LCA Commons process LCIs contains four NAICS categories and twenty-three NAICS subcategories.

Similarly, for Ecoinvent, applying the proposed method resulted in the identification and classification of 2766 process LCIs representing the production of 601 unique chemical entities. Classification into the ChemOnt taxonomy assigned materials into two kingdoms, eighteen superclasses, ninety-one classes, and 173 subclasses. The default industrial classification of the same Ecoinvent process LCIs contains six ISIC “divisions”, twelve ISIC “groups”, and twenty-three ISIC “classes”.

A significant increase in the number of taxonomic levels and classes was achieved by classifying LCI data representing the production of chemicals and materials available in the Federal LCA Commons and Ecoinvent into the ChemOnt chemical taxonomy according to the chemical structure of the product. The resulting environmental impact distributions exhibit reduced variance (spread) and greater specificity to the chemical structure of the constituent chemical entities, improving the utility of under-specification methods for filling LCA data gaps and identifying low-impact formulations in early-stage research and development of novel materials.

The median GWP impact values for each category in the ChemOnt taxonomy populated by Federal LCA Commons and Ecoinvent LCI data are shown using a color gradient in Figures 2 and 3 below, respectively, with the kingdom shown at the center and the chemical specificity increasing radially. The arc length of each tile corresponds to the number of unique process LCIs contained in that category. As the level of specificity increases, the number of processes contained in each category decreases. Classification of some chemical entities terminates at a lower hierarchical level than others, resulting in some categories that contain a larger number of processes than the sum of their subcategories.

**Figure 2:**
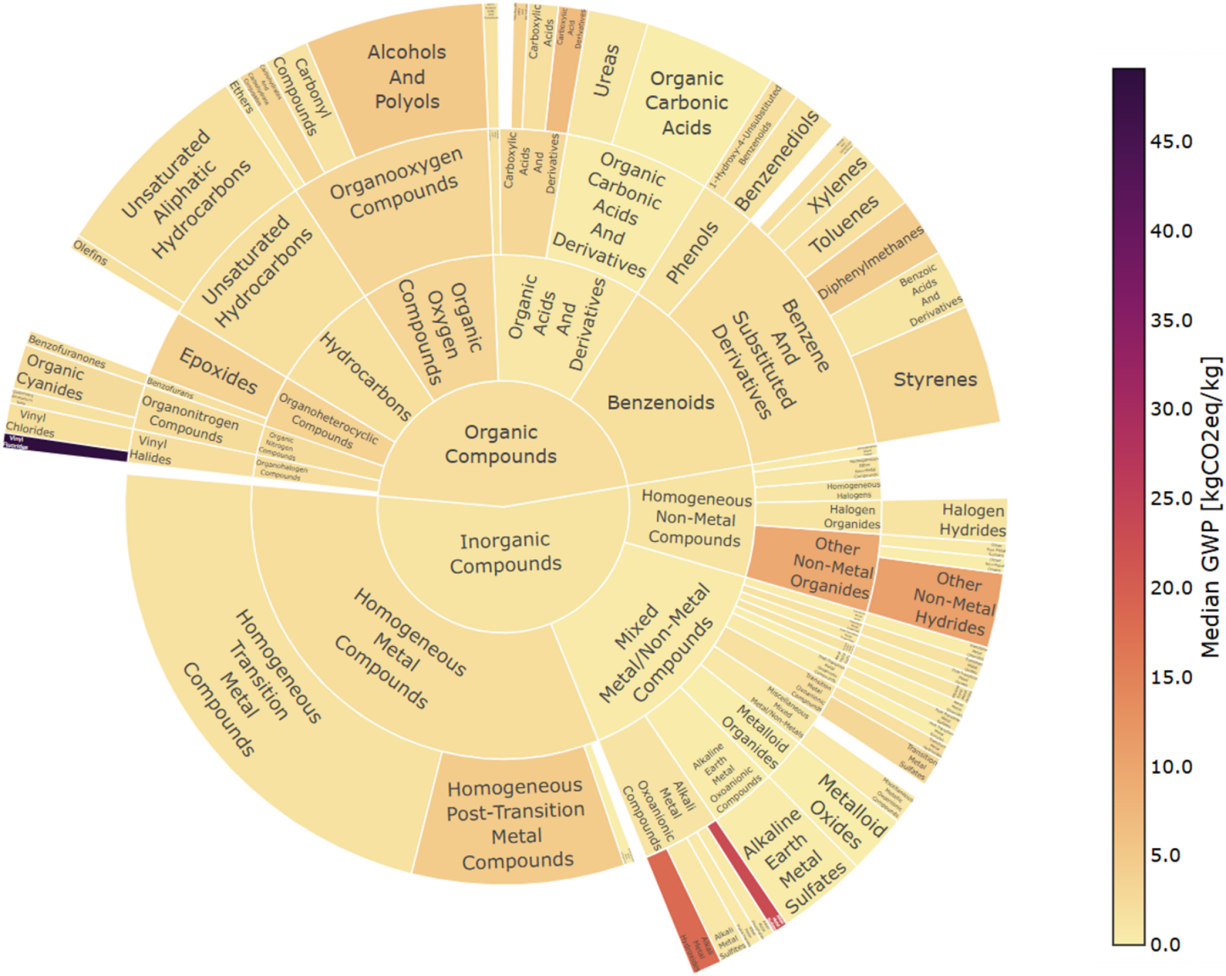
ClassyFire taxonomic classification of global warming potential (GWP) impact results for chemical entities with life cycle inventory (LCI) data available in the Federal LCA Commons. An interactive version of this figure can be downloaded as an HTML file from Figshare (DOI: https://doi.org/10.6084/m9.figshare.30996661).

**Figure 3:**
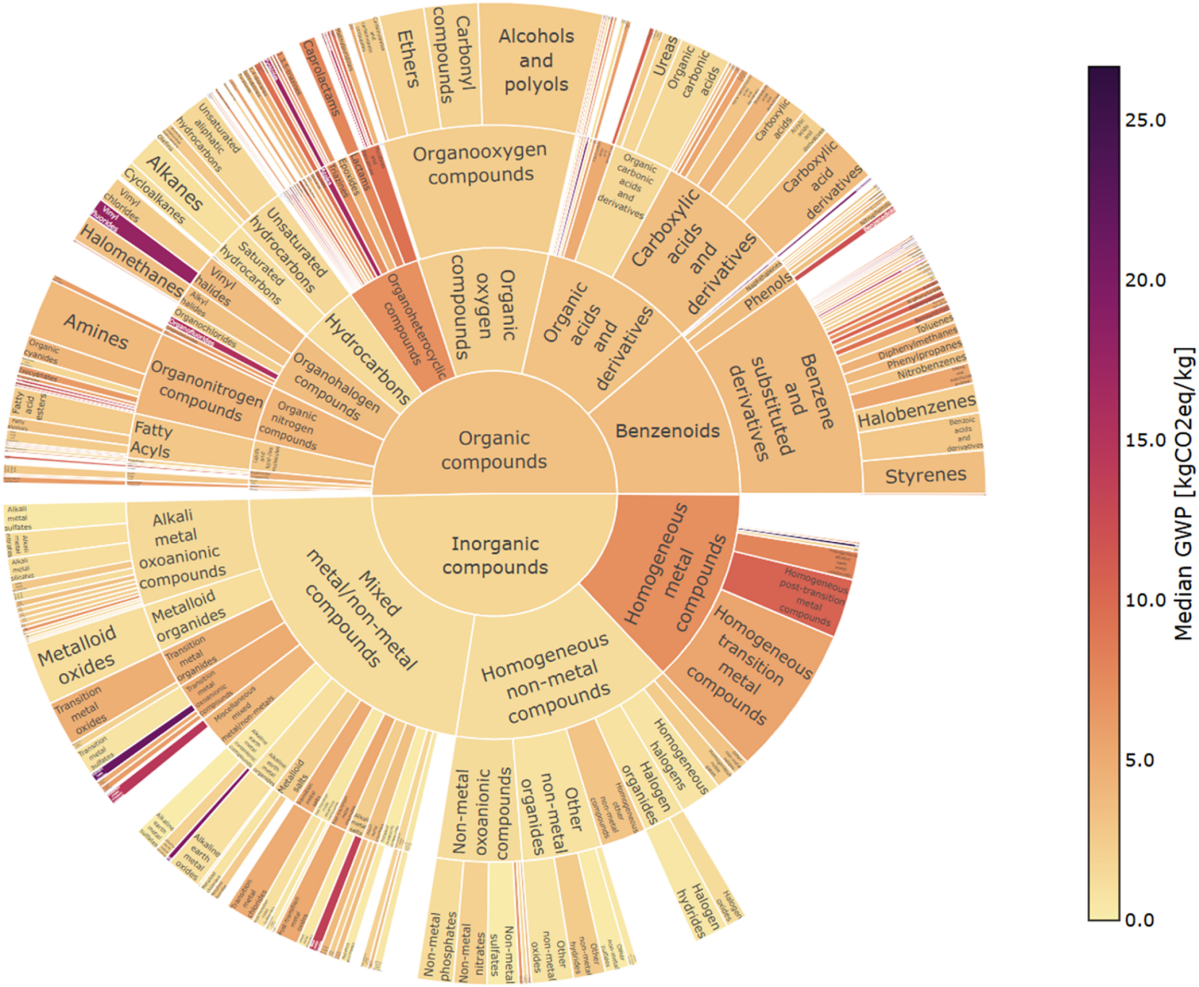
ClassyFire taxonomic classification of global warming potential (GWP) impact results for chemical entities with life cycle inventory (LCI) data available in Ecoinvent version 3.9.1. Actinide and transition metal thiosulfate categories are not shown.

The individual GWP results from the Federal LCA Commons process LCIs, classified into each category, are shown in Figure 4. Each point represents the GWP impact value calculated from the evaluation of the default product system generated for a process LCI for a material in that category. The presence of outliers can greatly skew the impact distribution of individual categories, and the variance of the distribution is reduced by removing outliers. Of the classified Federal LCA Commons data, one GWP outlier was excluded from Figures 2 and 4 (“Fuel grade uranium, at regional storage”). Of the classified Ecoinvent data, processes for the production of actinides and transition metal thiosulfates were identified as GWP outliers and are not shown in Figure 3.

**Figure 4:**
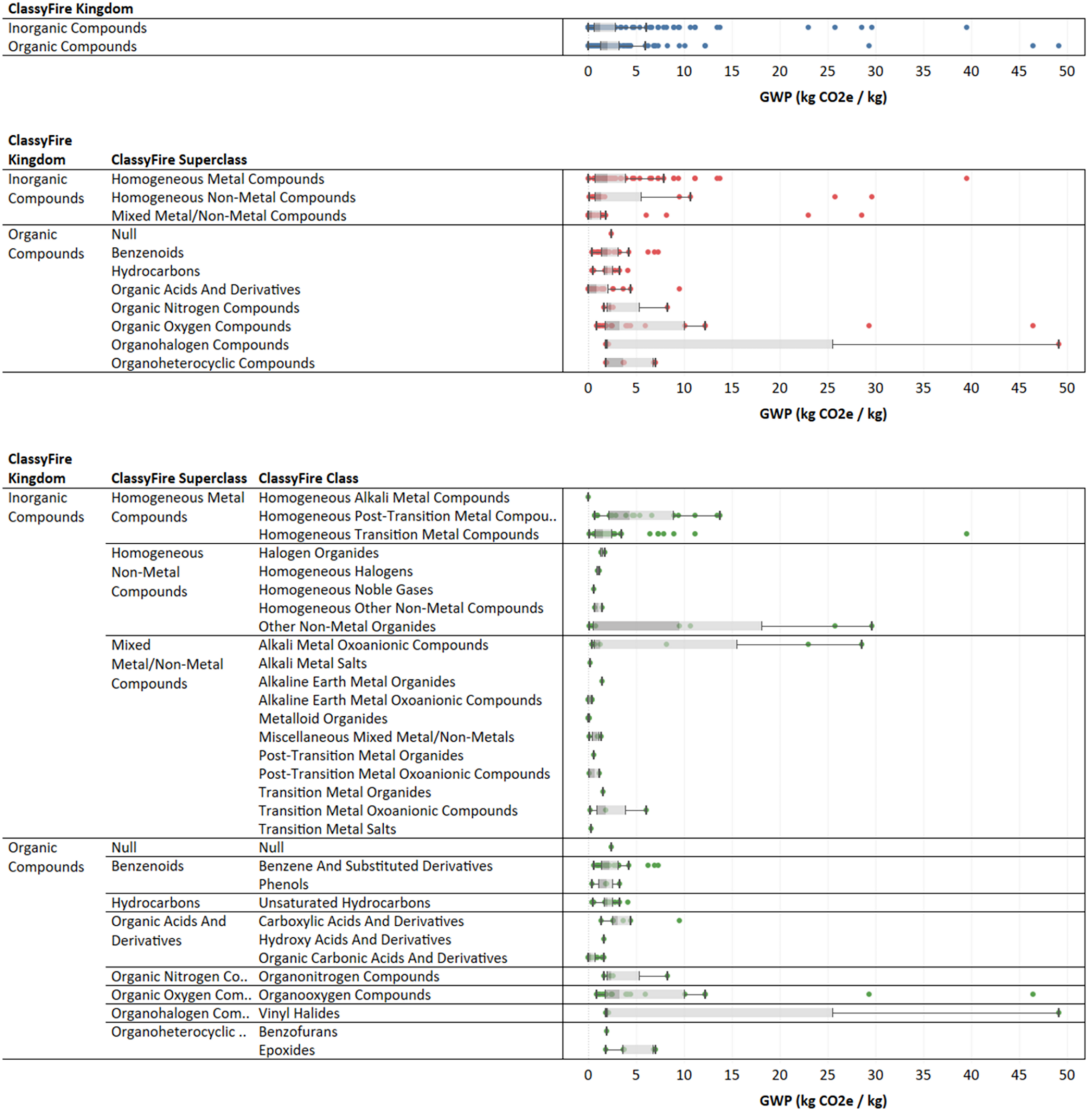
Partitioning of chemical and material GWP results in chemical kingdoms, superclasses, and classes using life cycle inventory (LCI) data available in the Federal LCA Commons.

Relative to the Federal LCA Commons database, Ecoinvent contains a significantly larger volume of process LCIs for a wider variety of unique chemical entities. Applying the proposed methods to the Ecoinvent database resulted in the identification of 2766 process LCIs for 601 unique chemical entities. Classification into the ChemOnt taxonomy assigned materials into two kingdoms, eighteen superclasses, ninety-one classes, and 173 subclasses. Specific environmental impact values are not shown for Ecoinvent data due to data privacy limitations. However, it is observed that the increased volume of data and number of chemical entities represented result in a larger number of categories populated with impact data at each level in the taxonomy. In turn, the environmental impacts of a wider variety of input materials and chemicals can be estimated to a higher level of specification. Additional levels of taxonomic classification are available for some chemical entities; however, only the kingdom through subclass levels of the ChemOnt taxonomy were included in this study because the majority of chemical entities were classified to at least the subclass level.

### 3.2 Case Studies

To demonstrate the method for filling data gaps in rapid screening LCAs for early-stage product design, a variety of potential input materials used in formulation and product development were rapidly evaluated for proxy environmental impact distributions using the methods described above and the LCI data available in the Federal LCA Commons.

Copper sheet is a commonly produced material used in various downstream applications; however, at the time of writing, there are no LCI data available in the Federal LCA Commons database representing the environmental impacts of producing copper sheet. On the other hand, environmental product declarations for copper sheet metal are available and provide a convenient source of publicly available data to validate proxy environmental impacts evaluated for copper sheet using the methods described above. In practice, proxy values should only be used if no other source of environmental impact values are available.

Similarly, tetrahydrofuran (THF) is a widely used solvent with LCI data available in the Ecoinvent database; however, no LCI data is available in the Federal LCA Commons database. Additional case studies including sodium silicate, poly­(vinyl alcohol) (PVA) and calcined clay (metakaolin) were selected to provide a greater variety of examples.

These five example material inputs, with corresponding chemical entity names, InChI Keys, and ChemOnt classifications summarized in Table 1, are presented as case studies to demonstrate the application of the proposed methods. The methods described in the previous sections were implemented to determine GWP impact distributions for the case study materials using open-source Federal LCA Commons data. The results for all materials except THF were compared with an EPD, and all except PVA were compared to the GWP impact result from the default product system generated using Ecoinvent data. No EPD was found for THF, and no Ecoinvent process was found for PVA.

**Table 1:** Case Study Materials and Their ChemOnt Classifications

Material input	Chemical entity name	InChI key	Kingdom	Superclass	Class	Subclass
copper sheet	copper	RYGMFSIKBFXOCR-UHFFFAOYSA-N	inorganic compounds	homogeneous metal compounds	homogeneous transition metal compounds	—
tetrahydrofuran	tetrahydrofuran	WYURNTSHIVDZCO-UHFFFAOYSA-N	organic compounds	organoheterocyclic compounds	tetrahydrofurans	—
sodium silicate	sodium silicate	NTHWMYGWWRZVTN-UHFFFAOYSA-N	inorganic compounds	mixed metal/nonmetal compounds	alkali metal oxoanionic compounds	alkali metal silicates
polyvinyl alcohol (PVA)	vinyl alcohol^a^	IMROMDMJAWUWLK-UHFFFAOYSA-N	organic compounds	organic oxygen compounds	organooxygen compounds	enols
calcined clay	metakaolinite	FTQIMBDDNTZVSO-UHFFFAOYSA-N	inorganic compounds	mixed metal/nonmetal compounds	metalloid oxoanionic compounds	metalloid aluminates

a Polymers that do not have a specified molecular weight and stereochemistry do not have a unique InChI key, and as such are classified according to the InChI key of their respective monomer unit.

For each example material input, the low (20th percentile), median, and high (80th percentile) GWP impact was determined using the material impact data classification and under-specification method detailed in Section 2. The resulting GWP impact distributions were then compared with the GWP impact value reported in an environmental product declaration (EPD) for the material under consideration. The GWP impact resulting from a corresponding process in the Ecoinvent database was also evaluated for comparison if available. The proxy GWP impact distribution from available Federal LCA Commons LCI data was also plotted along with the EPD or Ecoinvent impact results for comparison. It should be noted that detailed statistical methods have been implemented in past studies using probabilistic under-specification to fill LCA data gaps, and these methods provide a more robust under-specification analysis.^10,12,16^

The resulting estimated GWP impact distributions for each example input material using the available LCI data from the Federal LCA Commons are shown in Table 2. The most specific chemical classification with available environmental impact data is used for estimating environmental impacts. This ensures the proxy environmental impact distribution derives from known environmental impacts of other materials with the most similar chemical composition and structure.

**Table 2:** Proxy GWP Impact Distributions Compared to EPD GWP Values and Ecoinvent Default Product System GWP Values (Depending on Data Availability)

Material input	Federal LCA Commons proxy distributions			Environmental product declaration impact values				Ecoinvent process	
	ClassyFire chemical entity name	Kingdom GWP median (20th%, 80th%) [kgCO2eq/kg]	Superclass GWP median (20th%, 80th%) [kgCO2eq/kg]	Class GWP median (20th%, 80th%) [kgCO2eq/kg]	Subclass GWP median (20th%, 80th%) [kgCO2eq/kg]	EPD GWP (A1–A3) [kgCO2eq/kg]	EPD reference (link)	Ecoinvent process name	Ecoinvent process UUID
copper sheet	copper	1.318 (0.538, 6.260)	2.126 (0.699, 6.973)	1.565 (0.696, 3.229)	N/A	2.72	Copper Development Association: LCA of Copper Sheet^34^	market for copper, anode	12ab4069–28 fc-44f1–86af-c9aacc1f81ae
tetrahydrofuran	tetrahydrofuran	2.036 (1.014, 3.737)	2.122 (2.205, 3.859)	no data available	N/A	no EPD Available	N/A	market for tetrahydrofuran	e699f8a1–4918–42d9–855f-bdcce1c658cb
sodium silicate	sodium silicate	1.318 (0.538, 6.260)	0.356 (0.041, 1.405)	1.188 (0.572, 19.996)	no data available	0.408	Prochin Italia Sodium Silicates^35^	market for sodium silicate, solid	119ea421–9862–47a6-b34b-843879093024
polyvinyl alcohol (PVA)	vinyl alcohol	2.036 (1.014, 3.737)	3.200 (1.623, 11.333)	3.200 (1.623, 11.333)	no data available	6.33	Sinopec Polyvinyl Alcohol Fibers^36^	N/A	N/A
calcined clay	metakaolinite	1.318 (0.538, 6.260)	0.356 (0.041, 1.405)	no data available	no data available	0.29	Christy Minerals Dynapoz (Metakaolin)^37^	market for calcined clay	d3411723–9907–49f9–8f21-f3cf410ffc61

For copper sheet as an example, the most specific chemical category assigned is *homogeneous transition metal compounds*, at the Class level. The median GWP impact corresponding to this class is 1.565 kgCO2eq/kg, with 20th and 80th percentile values of 0.696 and 3.229 kgCO2eq/kg, respectively. For comparison, the benchmark GWP impact of copper sheet production (LCA stages A1–A3) reported by the Copper Development Association is 2.72 kgCO2eq/kg, which is within the 20th to 80th percentile range.^34^

For sodium silicate, poly­(vinyl alcohol), and calcined clay, the corresponding ChemOnt subclass had no LCI data for any constituent entities available in the Federal LCA commons. Utilization of a database with a larger number of chemical entities represented, such as Ecoinvent, results in a larger number of categories populated with the LCI data at each taxonomic level. Larger LCI databases, in turn, enable the selection of proxy environmental impact distributions at more specific taxonomic levels for a wider variety of chemical and material inputs under consideration.

In order not to publicly release Ecoinvent data, we anonymized the four materials with Ecoinvent data and compared them with the results above. Results from the comparison of proxy GWP impact values to impact values determined from Ecoinvent processes are shown below in Table 3. For some materials, the median GWP proxy value is closer to the impact result obtained from the Ecoinvent process, while for other materials, the 80th percentile proxy value is closer.

**Table 3:** Results of Comparing Proxy GWP Impact Values Determined Above to Impact Values Determined from Processes Available in Ecoinvent

Material	Ecoinvent process includes impact of transport to user	|Ecoinvent % difference| from Median Federal LCA Commons GWP proxy value	|Ecoinvent % difference| from high (80th%) Federal LCA Commons GWP proxy value
A	yes	285%	87%
B	yes	188%	59%
C	yes	29%	96%
D	no	24%	81%

The GWP impact distributions for each ChemOnt kingdom, superclass, class, and subclass applicable to the material inputs considered in Table 2, generated from Federal LCA Commons LCI data, are shown in Figure 5. Uranium, indium, and mercury data points are not included in this figure. As specificity increases from kingdom to subclass, the variance of GWP impact distribution typically decreases. Removing outliers for statistical calculations produces proxy impact estimates with lower variance. However, this approach risks removing data points that may be relevant for estimation of proxy environmental impacts, especially if the input material lacking data is a specialty material that is better represented by one of the available outliers. Whether outliers should be included or removed depends on the LCI data available and the target compound for which environmental impacts are being estimated. The screening LCA process is iterative, and further analysis of data sources may be conducted for any identified environmental impact hotspots in the subsequent screening LCA iteration.

**Figure 5:**
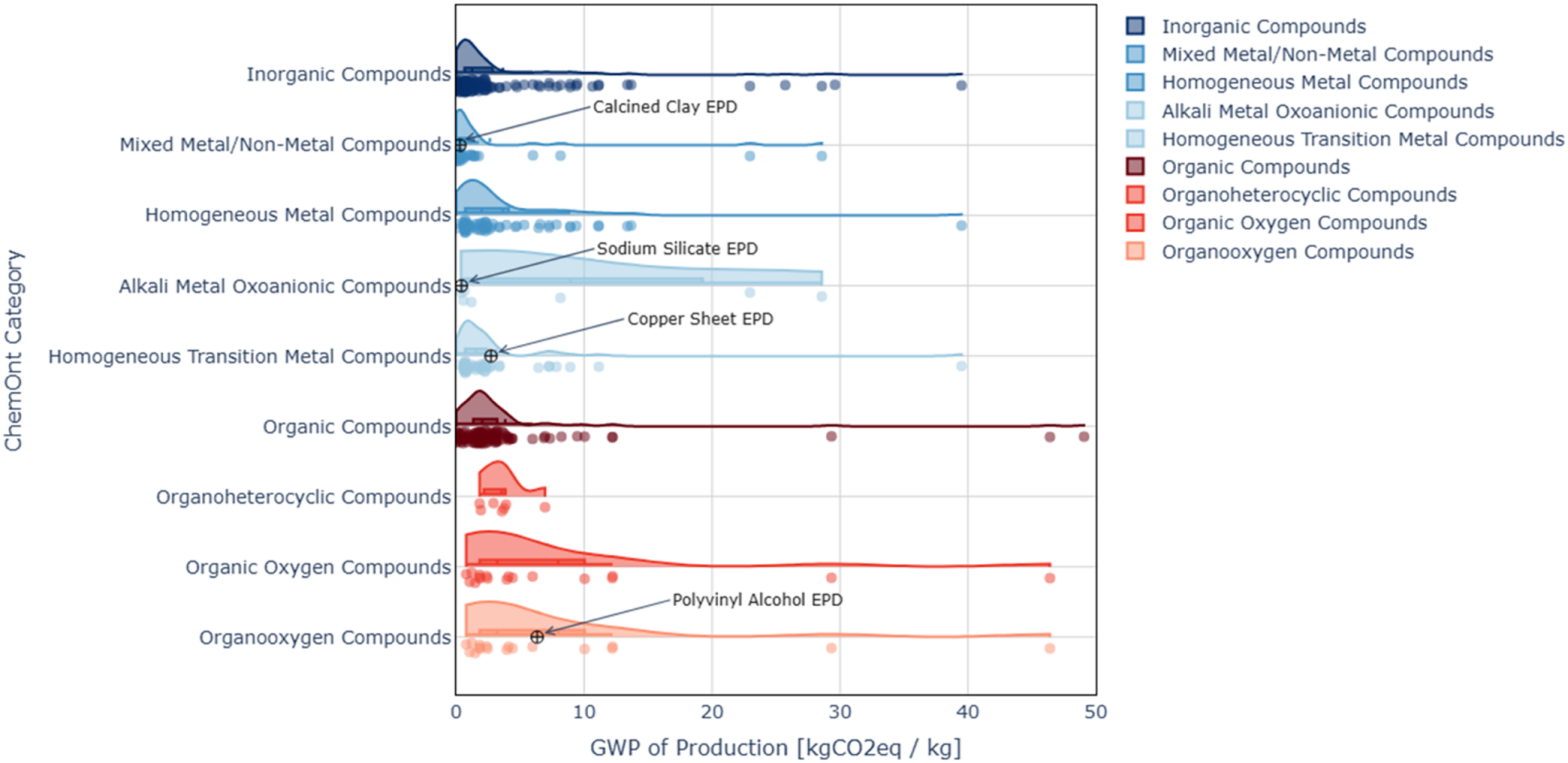
Evaluation of proxy global warming potential (GWP) distributions, for example, material inputs. GWP impact values reported by EPDs for case study materials are shown in the most specific corresponding ChemOnt category with available data.

For all example input materials considered in Table 2, with the exception of tetrahydrofuran, the 80th percentile GWP proxy impact estimate successfully bounds the GWP impact reported in the reviewed EPD or resulted from the comparable Ecoinvent process. For poly­(vinyl alcohol), the EPD GWP value falls between the third quartile and the maximum proxy GWP values. For tetrahydrofuran, the proxy GWP impact distribution does not bound the Ecoinvent GWP value. In fact, the Ecoinvent GWP value for tetrahydrofuran is greater than the largest GWP value corresponding to any organic compound in the Federal LCA Commons. This highlights the sensitivity of the proposed methods to the quality and availability of LCI data used to generate proxy impact distributions.

## 4 Discussion

Previous studies have demonstrated the applicability of under-specification methods to screening LCA in early-stage material and product development. However, these methods lacked transparency and reproducibility in the taxonomy schemas used for material classification. Data gaps for traditional materials produced in large volumes have been reduced with the recent growth in volume and availability of LCA data. As the data gaps for the most common materials are filled, the remaining hard-to-fill LCA data gaps are most likely to be specialty materials and chemicals produced in smaller volumes.

By utilizing a structure-based chemical taxonomy for under-specification methods, a higher degree of granularity for material and chemical groups can be achieved relative to industrial classification systems. In addition, classification of materials and chemicals based on chemical structure is more objective and reproducible compared to industrial category definitions, because any single material input may exhibit various industrial uses. The methods proposed herein build on the assumption that materials with similar chemical compositions and structures exhibit similar environmental impacts from production. This assumption is logically consistent with the observation that materials having similar chemical compositions and structures are typically derived from similar raw materials and undergo similar chemical processing.

For a more robust understanding of the range of possible environmental impacts likely resulting from production of the target chemical entity, the statistical distribution of environmental impact values could be used to conduct sensitivity analyses or Monte Carlo Simulations. This methodology can enable developers of novel materials to identify environmental impact hotspots and distinguish material formulations that are likely to result in significantly lower environmental impacts early in development, even when data gaps exist. Proxy environmental impact values should be used in only LCA applications if no other source of LCI or environmental impact data is available.

One limitation of this method is the inability to classify composite materials and materials that are mixtures of numerous chemical entities into a single chemical class. Only materials and chemicals returning a valid InChI key or SMILES structure from the chemical name search were included in this study, potentially excluding data for raw or composite materials, which could be classifiable by the major component. Composite materials or raw materials with significant concentrations of impurities may be classified according to their major component within an established purity threshold; however, material purity was not considered in this study. While the methods in this article are not applicable to high-volume composite construction materials such as concrete, industrial classification systems are useful for applying under-specification methods to such materials. Future studies may investigate methods to increase the granularity and depth of industrial classification systems or other material taxonomies, which include composite materials, for example, by classifying concrete LCA results by compressive strength or cement content. Polymers with no specified molecular weight and stereochemistry do not have a unique InChI key or SMILES structure; therefore, polymers were classified according to the InChI key of their respective monomer unit without accounting for molecular weight or stereochemistry.

Another limitation is that the chemical structure may not always be the best indicator of environmental impacts for producing a specific material or chemical. For instance, raw mineral materials with radically different chemical compositions can exhibit similar material properties and undergo similar processing steps, in which case environmental impacts may be adequately estimated via under-specification in an industrial classification system. Classification by chemical synthesis methods or key unit processes may better resolve environmental impacts compared with chemical structure classification. The authors are not currently aware of any existing studies that have compared the homogeneity of environmental impact distributions for differing material classification taxonomies.

Increasing the depth (number of levels) and granularity of the taxonomy in under-specification methods can also result in increased homogeneity of environmental impacts for material and chemical categories. In turn, this could make the under-specification method more effective for statistically differentiating the environmental impacts of comparable product designs. In other words, reducing the variance of the predicted result distributions by increasing the specificity of material categories could lead to less overlap of the resulting product impact distributions and greater confidence in distinguishing impact results of competing designs.

The case study analysis in this study is simplistic relative to previous implementations of probabilistic under-specification methods in the LCA literature. In this study, the median and 80th percentile GWP values of identified proxy distributions were selected as deterministic proxy values at only one specification level and compared to available EPD and Ecoinvent GWP results. Combining the material classification methods proposed herein with advanced probabilistic methods such as Monte Carlo simulations and probabilistic triage as demonstrated in previous under-specification literature could better investigate the potential of material classification based on chemical structure to resolve environmental impact distributions of competing designs.^9,10,12,16,17^ Additionally, investigating the variance of resulting impact distributions at different specification levels such as demonstrated in past studies could enable identification of critical levels of specification needed to resolve impact distributions of competing designs.^6,10,12^ Future studies may also consider incorporating the production volume of materials to calculate weighted environmental impact distributions.^14^ In addition, the framework proposed herein could be expanded with more robust analyses of data quality, sensitivity analyses, or other statistical analyses. Beyond the production stage environmental impacts, probabilistic under-specification methods could also be applied to predict environmental impact distributions for end-of-life processing and disposal of chemical entities.^4,8,38^ Although not discussed in this study, these methods may also be used to estimate quantities of individual emissions resulting from the production of a chemical entity by utilizing LCI data prior to characterization with an LCIA method.

The methods proposed herein are intended as a useful framework for the estimation of proxy environmental impacts in early-stage screening LCAs. The utility and accuracy of this method depend on the volume and quality of environmental impact data used to populate the material taxonomy. As the volume and quality of LCA data continue to grow internationally, these methods can be used to maximize the utility of available data to bridge data gaps for specialty chemicals and materials in early-stage research and development. The authors find that utilizing these methods and selecting the 80th percentile impact value of the most specific corresponding material group populated with impact data can be a reasonable conservative method to identify a deterministic proxy environmental impact value when necessary, and that varying between 20th and 80th percentile values can be an effective method for conducting a sensitivity analysis when time, resource, or expertise constraints prevent further data collection or in-depth statistical analyses. If the contributions of the proxy impacts to the overall LCA result are significant, then practitioners know that further data collection efforts for the data gap are justified and are encouraged to identify a more specific and representative LCI data set. Utilizing this proxy method can enable more consistent and reproducible methods for filling data gaps in LCA studies, especially when the alternative strategies may be limited to arbitrary choices of proxy data sets or omitting the data.

## Supplementary Material



## Notes

A spreadsheet file containing formulas for automatic InChI key search and Federal LCA Commons environmental impact data and distributions by chemical class (XLSX)
